# Supplementary material for: EPY001, a Novel Monoclonal Antibody Against Pseudomonas aeruginosa Targeting OprF
Source: Int J Mol Sci. 2025 Oct 25;26(21):10380. doi: 10.3390/ijms262110380 (PMC12609887; doi:10.3390/ijms262110380)
Supplement: Supplementary file 1 [file ijms-26-10380-s001.zip › ijms-3937590-supplementary.pdf]

Supplementary Materials:

Table S1. Bacterial Isolates Employed in This Study.

Strains	Species	Comments	References
CHA	*Pseudomonas aeruginosa*	Isolated in 1990 from the broncho-pulmonary tract of a cystic fibrosis patient at Grenoble University Hospital	[1]
CHAΔOprF	*Pseudomonas aeruginosa*	obtained by homologous recombination with an OprF fragment containing a gentamycin acetyltransferase (Sm) cassette	Pr Jean-Luc Lenormand
H103	*Pseudomonas aeruginosa*	PAO1 wild-type prototroph	[2]
H636 (H103ΔOprF)	*Pseudomonas aeruginosa*	obtained by homologous recombination with an OprF fragment containing a streptomycin (Sm) cassette	[2]
51.3B	*Pseudomonas aeruginosa*	Clinical Isolate from Grenoble Grenoble University Hospital, associated with lung infection	Caroline Plazy
108.1	*Pseudomonas aeruginosa*	Clinical Isolate from Grenoble Grenoble University Hospital, associated with lung infection	Caroline Plazy
PA7ET	*Pseudomonas aeruginosa*	Multidrug-resistant clinical isolate from Grenoble University Hospital, associated with acute pulmonary infection	Epynext Therapeutics
PA10ET	*Pseudomonas aeruginosa*	Multidrug-resistant clinical isolate from Grenoble University Hospital, associated with acute pulmonary infection	Epynext Therapeutics
PA12ET	*Pseudomonas aeruginosa*	Multidrug-resistant clinical isolate from Grenoble University Hospital, associated with	Epynext Therapeutics

		acute pulmonary infection	
PA16ET	*Pseudomonas aeruginosa*	Multidrug-resistant clinical isolate from Grenoble University Hospital, associated with acute pulmonary infection	Epynext Therapeutics
E. coli	*Escherichia coli*	Multidrug-resistant clinical isolate from Grenoble University Hospital, associated with acute pulmonary infection	Epynext Therapeutics
A. baumannii	*Acinetobacter baumannii*	Multidrug-resistant clinical isolate from Grenoble University Hospital, associated with acute pulmonary infection	Epynext Therapeutics

Table S2. WT and mutated OprF sequences in the plasmid pIVEX2.4d. Sequence of the pIVEX2.4d plasmid can be accessed on the Biotech Rabbit website.

Names	Sequences
OprF WT	MSGSHHHHHHSSGIEGRGRLIKHMKLKNTLGVVIGSLVAASAMNAFAQGQNSVEIEAFG<br>KRYFTDSVRNMKNADLYGGSIGYFLTDDVELALSYGEYHDVRGTYETGNKKVHGNLTSL<br>DAIYHFGTPGVGLRPYVSAGLAHQNITNINSDSQGRQQMTMANIGAGLKYYFTENFFAKA<br>SLDGQYGLEKRDNGHQGEWMAGLGVGFNFGGSKAAPAPEPVADVCSDSDNDGVCDNV<br>DKCPDTPANVTVDANGCPAVAEVVRVQLDVKFDFDKSKVKENSYADIKNLADFMKQYP<br>STSTTVEGHTDSVGTDAYNQKLSERRANAVRDVLVNEYGVEGGRVNAVGYGESRPVAD<br>NATAEGRAINRRVEAEVEAEAK
OprF mel2	MSGSHHHHHHSSGIEGRGRLIKHMKLKNTLGVVIGSLVAASAMNAFAQGQNSVEIEAFG<br>KRYFTDSVRNMKNADLYGGSIGYFLTDDVELALSYGEYHDVRGTAATGAKAVHGNLTS<br>LDAIYHFGTPGVGLRPYVSAGLAHQNITNINSDSQGRQQMTMANIGAGLKYYFTENFFAK<br>ASLDGQYGLEKRDNGHQGEWMAGLGVGFNFGGSKAAPAPEPVADVCSDSDNDGVCDN<br>VDKCPDTPANVTVDANGCPAVAEVVRVQLDVKFDFDKSKVKENSYADIKNLADFMKQY<br>PSTSTTVEGHTDSVGTDAYNQKLSERRANAVRDVLVNEYGVEGGRVNAVGYGESRPVA<br>DNATAEGRAINRRVEAEVEAEAK
OprF mel4	MSGSHHHHHHSSGIEGRGRLIKHMKLKNTLGVVIGSLVAASAMNAFAQGQNSVEIEAFG<br>KRYFTDSVRNMKNADLYGGSIGYFLTDDVELALSYGEYHDVRGTYETGNKKVHGNLTSL<br>DAIYHFGTPGVGLRPYVSAGLAHQNITNINSDSQGRQQMTMANIGAGLKYYFTENFFAKA<br>SLDGQYGLEKRANGAAGAWMAGLGVGFNFGGSKAAPAPEPVADVCSDSDNDGVCDNV<br>DKCPDTPANVTVDANGCPAVAEVVRVQLDVKFDFDKSKVKENSYADIKNLADFMKQYP<br>STSTTVEGHTDSVGTDAYNQKLSERRANAVRDVLVNEYGVEGGRVNAVGYGESRPVAD<br>NATAEGRAINRRVEAEVEAEAK

OprF mel5	MSGSHHHHHHSSGIEGRGRLIKHMKLKNTLGVVIGSLVAASAMNAFAQGQNSVEIEAFG<br>KRYFTDSVRNMKNADLYGGSIGYFLTDDVELALSYGEYHDVRGTYETGNKKVHGNLTSL<br>DAIYHFGTPGVGLRPYVSAGLAHQNITNINSDSQGRQQMTMANIGAGLKYYFTENFFAKA<br>SLDGQYGLEKRDNGHQGEWMAGLGVGFNFGGSKAAPAPEPVADVCSASANDGACDAV<br>DKCPDTPANVTVDANGCPAVAEVVRVQLDVKFDFDKSKVKENSYADIKNLADFMKQYP<br>STSTTVEGHTDSVGTDAYNQKLSERRANAVRDVLVNEYGVEGGRVNAVGYGESRPVAD<br>NATAEGRAINRRVEAEVEAEAK
OprF mel6	MSGSHHHHHHSSGIEGRGRLIKHMKLKNTLGVVIGSLVAASAMNAFAQGQNSVEIEAFG<br>KRYFTDSVRNMKNADLYGGSIGYFLTDDVELALSYGEYHDVRGTYETGNKKVHGNLTSL<br>DAIYHFGTPGVGLRPYVSAGLAHQNITNINSDSQGRQQMTMANIGAGLKYYFTENFFAKA<br>SLDGQYGLEKRDNGHQGEWMAGLGVGFNFGGSKAAPAPEPVADVCSDSDNDGVCDNV<br>DKCPDTPANVTVDANGCPAVAEVVRVQLDVKFDFDKSAVAAASYADIKNLADFMKQYP<br>STSTTVEGHTDSVGTDAYNQKLSERRANAVRDVLVNEYGVEGGRVNAVGYGESRPVAD<br>NATAEGRAINRRVEAEVEAEAK
OprF mel7	MSGSHHHHHHSSGIEGRGRLIKHMKLKNTLGVVIGSLVAASAMNAFAQGQNSVEIEAFG<br>KRYFTDSVRNMKNADLYGGSIGYFLTDDVELALSYGEYHDVRGTYETGNKKVHGNLTSL<br>DAIYHFGTPGVGLRPYVSAGLAHQNITNINSDSQGRQQMTMANIGAGLKYYFTENFFAKA<br>SLDGQYGLEKRDNGHQGEWMAGLGVGFNFGGSKAAPAPEPVADVCSDSDNDGVCDNV<br>DKCPDTPANVTVDANGCPAVAEVVRVQLDVKFDFDKSKVKENSYADIKNLADFMKQYP<br>STSTTVEGHTDSVGTDAAAQALSARRANAVRDVLVNEYGVEGGRVNAVGYGESRPVAD<br>NATAEGRAINRRVEAEVEAEAK
OprF mel8	MSGSHHHHHHSSGIEGRGRLIKHMKLKNTLGVVIGSLVAASAMNAFAQGQNSVEIEAFG<br>KRYFTDSVRNMKNADLYGGSIGYFLTDDVELALSYGEYHDVRGTYETGNKKVHGNLTSL<br>DAIYHFGTPGVGLRPYVSAGLAHQNITNINSDSQGRQQMTMANIGAGLKYYFTENFFAKA<br>SLDGQYGLEKRDNGHQGEWMAGLGVGFNFGGSKAAPAPEPVADVCSDSDNDGVCDNV<br>DKCPDTPANVTVDANGCPAVAEVVRVQLDVKFDFDKSKVKENSYADIKNLADFMKQYP<br>STSTTVEGHTDSVGTDAYNQKLSERRANAVRDVLVNEYGVEGGRVNAVGYGESRPVAD<br>NATAEGRAINRRVEAEVEAEAK

Table S3. Amino acid sequences of antibodies including secretion signal peptide.

Antibody	Variable Light chain	Variable Heavy chain
Anti-lysozyme IgG control	DIVLTQSPASLSASVGETVTITCRA<br>SGNIHNYLAWYQQKQGKSPQLLV<br>YYTTTLADGVPSRFSGSGSGTQYSL<br>KINSLQPEDFGSYYCQHFWSTPRT<br>FGGGTKLEIKRADAAPTVSIFPPSS<br>EQLTSGGASVVCFLNNFYPKDINV<br>KWKIDGSERQNGVLNSWTDQDS<br>KDSTYSMSSTLTLTKDEYERHNSY<br>TCEATHKTSTSPIVKSFNRNEC	QVQLQESGPGLVAPSQSLSITCTVSGFSLTG<br>YGVNWVRQPPGKGLEWLGMIWGDGNTD<br>YNSALKSRLSISKDNSKSQVFLKMNSLHTD<br>DTARYYCARERDYRLDYWGQGTTLTVSSA<br>KTTAPSVYPLAPVCGDTTGSSVTLGCLVKG<br>YFPEPVTLTWNSGSLSSGVHTFPAVLQSDLY<br>TLSSSVTVTSSTWPSQSITCNVAHPASSTKV<br>DKKIEPRGPTIKPCPPCKCPAPNLLGGPSVFI<br>FPPKIKDVLMISLSPIVTCVVVDVSEDDPDV<br>QISWFVNNVEVHTAQTQTHREDYNSTLRV<br>VSALPIQHQDWMSGKEFKCKVNNKDLPAP<br>IERTISKPKGSVRAPQVYVLPPPEEEMTKKQ

	VTLTCMVTDFMPEDIYVEWTNNGKTELNY<br>KNTEPVLDSDGSYFMYSKLRVEKKNWVER<br>NSYSCSVVHEGLHNHHTTKSFSRTPGK

References Supplementary Materials:

[1] B. Toussaint, I. Delic-Attree, et P. M. Vignais, « Pseudomonas aeruginosa contains an IHF-like protein that binds to the algD promoter », *Biochem. Biophys. Res. Commun.*, vol. 196, nº 1, p. 416-421, oct. **1993**, doi: 10.1006/bbrc.1993.2265.

[2] W. A. Woodruff et R. E. Hancock, « Construction and characterization of Pseudomonas aeruginosa protein F-deficient mutants after in vitro and in vivo insertion mutagenesis of the cloned gene », *J. Bacteriol.*, vol. 170, nº 6, p. 2592-2598, juin **1988**, doi: 10.1128/jb.170.6.2592-2598.1988.
